# Not Just the Patient: Addressing Somatic Vigilance and Fear of Illness in a Family Caregiver – A Case-Based CBT Nursing Approach

**DOI:** 10.1192/j.eurpsy.2026.10796

**Published:** 2026-07-31

**Authors:** A. Argyriadi, S. Kotrotsiou, E. Kotrotsiou, A. Argyriadis

**Affiliations:** 1 Frederick University, Nicosia, Cyprus; 2 University of Patras, Patra; 3 Hellenic Mediterranean University, Evidence based Health, Education and Clinical Protocols Laboratory, Heraklion, Greece

## Abstract

**Introduction:**

While oncology care traditionally emphasizes addressing the physical and emotional needs of patients, the profound psychological burden carried by family caregivers often remains underexplored. This under-recognized phenomenon can lead caregivers to misinterpret benign bodily sensations as signs of serious illness, mirroring features of illness anxiety disorder and compounding their distress.

**Objectives:**

The objectives of this study are to explore the psychological impact of caregiving on perceived personal health vulnerability, with particular attention to how the continuous stress of supporting a loved one with cancer may heighten self-perceptions of fragility. It further seeks to examine the emergence of somatic vigilance and catastrophic health beliefs among caregivers, patterns that often intensify anxiety and mimic features of illness-related disorders. By highlighting these dynamics, the study aims to demonstrate the pivotal role of mental health nurses in delivering evidence-based, CBT-informed interventions that address such fears and foster resilience. Finally, it intends to provide a practical framework for systematic screening and early intervention in nursing practice, offering a pathway to more holistic and preventive approaches in caregiver support.

**Methods:**

A 45-year-old female caregiver of a patient with metastatic colon cancer presented with persistent fear of developing cancer herself. The case was evaluated using clinical interviews, nursing observation, and validated tools (e.g., the Health Anxiety Inventory). A CBT-informed nursing protocol was applied over 6 sessions, emphasizing on Psychoeducation on cognitive distortions related to health anxiety, Behavioral experiments and exposure to avoided situations (e.g., routine medical checkups), Cognitive restructuring of catastrophic interpretations of bodily sensations and Grounding techniques and emotion-focused regulation strategies for panic episodes.

**Results:**

Over the course of the six-week protocol, the caregiver exhibited a marked reduction in health-checking behaviors, alongside a strengthened capacity to tolerate uncertainty and a renewed commitment to self-care routines. Quantitative assessment reflected these gains, with pre- to post-intervention scores on the Health Anxiety Inventory (HAI) declining substantially from 36 to 17, indicating a clinically meaningful improvement.

**Conclusions:**

This case illustrates the vital role of mental health nurses in detecting and addressing CBT-relevant psychological patterns not only in patients, but in their caregivers. A structured, CBT-informed nursing approach can mitigate maladaptive fear, reduce caregiver burden, and enhance overall resilience in the cancer care context.

**Disclosure of Interest:**

None Declared

